# Curcumin exerts anti-inflammatory and vasoprotective effects through amelioration of NFAT-dependent endothelin-1 production in mice with acute Chagas cardiomyopathy

**DOI:** 10.1590/0074-02760180171

**Published:** 2018-07-16

**Authors:** Matías Hernández, Susana Wicz, Miguel H Santamaría, Ricardo S Corral

**Affiliations:** 1 Universidad Nacional de San Luis, Facultad de Química, Bioquímica y Farmacia, Laboratorio de Biomedicina Molecular, San Luis, Argentina; 2 Centro de Estudios Metabólicos, Laboratorio de Biología Experimental, Santander, Spain; 3 Hospital de Niños Dr Ricardo Gutiérrez, Instituto Multidisciplinario de Investigaciones en Patologías Pediátricas, Servicio de Parasitología-Chagas, Ciudad Autónoma de Buenos Aires, Argentina

**Keywords:** Chagas disease, cardiomyopathy, curcumin, endothelin-1, vasculitis

## Abstract

**OBJECTIVE:**

To evaluate the effects of oral therapy with Cur on *T. cruzi*-mediated cardiovasculopathy in acutely infected mice and analyse the *in vitro* response of parasite-infected human microvascular endothelial cells treated with this phytochemical.

**METHODS:**

Inflammation of heart vessels from Cur-treated and untreated infected mice were analysed by histology, with benznidazole (Bz) as the reference compound. Parasitaemia was monitored by the direct method. Capillary permeability was visualised by Evans-blue assay. Myocardial *ET-1*, *IL-6*, and *TNF-α* mRNA expressions were measured by quantitative reverse transcription polymerase chain reaction (qRT-PCR). Microvascular endothelial HMEC-1 cells were infected *in vitro* with or without addition of Cur or Bz. Induction of the Ca^2+^/NFAT pathway was assessed by fluorometry, immunoblotting, and reporter assay.

**FINDINGS:**

Oral Cur therapy of recently infected mice reduced inflammatory cell infiltration of myocardial arteries without lowering parasite levels. Compared to that of the phosphate-buffered saline-receiving group, hearts from Cur-treated mice showed significantly decreased vessel inflammation scores (p < 0.001), vascular permeabilities (p < 0.001), and levels of *IL-6*/*TNF-α* (p < 0.01) and *ET-1* (p < 0.05) mRNA. Moreover, Cur significantly (p < 0.05 for transcript; p < 0.01 for peptide) downregulated ET-1 secretion from infected HMEC-1 cells. Remarkably, Cur addition significantly (p < 0.05 at 27.0 μM) interfered with *T. cruzi*-dependent activation of the Ca^2+^/NFATc1 signalling pathway that promotes generation of inflammatory agents in HMEC-1 cells.

**CONCLUSIONS:**

Oral treatment with Cur dampens cardiovasculopathy in acute Chagas mice. Cur impairs the Ca^2+^/NFATc1-regulated release of ET-1 from *T. cruzi*-infected vascular endothelium. These findings identify new perspectives for exploring the potential of Cur-based interventions to ameliorate Chagas heart disease.

Chagas disease, caused by infection with the protozoan parasite *Trypanosoma cruzi*, is a major cause of acute myocarditis and chronic cardiomyopathy in endemic areas of Latin America. The high rate of migration toward non-endemic countries has spread the infection to other continents. This human parasitosis is characterised by acute and chronic phases. The mortality rate of early Chagas disease is 5-10%, usually due to severe myocarditis or meningoencephalitis. Following initial infection, most patients enter into an asymptomatic, indeterminate stage, which lasts throughout life in the majority of hosts. The remaining 30% of infected individuals develop digestive or, more frequently, cardiac complications that may lead to stroke and sudden death, typically years or decades after initial infection.

Chronic Chagas heart disease manifests itself as a dilated congestive cardiomyopathy associated with long-term inflammation and fibrosis, cardiac hypertrophy and thromboembolic events. Heart failure in chronically infected hosts is the culmination of a pathologic process that begins during the acute phase of infection.^1^ ^,^ ^2^ The precise aetiology of this condition still remains elusive. To date, data from studies in animal models and humans support a multifactorial hypothesis comprising four central pathogenic mechanisms of *T. cruzi*-elicited cardiomyopathy: (i) parasite-dependent heart damage; (ii) immune-mediated myocardial injury; (iii) cardiac dysautonomia; and (iv) vascular dysfunction.^3^

Microvascular disturbances that modify blood distribution in the cardiac muscle of Chagas patients contribute to cardiomyocyte destruction and ischaemia. This compromised microcirculation involves endothelial alterations, vasospasm, reduced blood flow, and focal ischaemia. Cardiovascular production of vasoactive mediators has been implicated in the pathogenesis of the vasculopathy seen in Chagas myocarditis.^4^ One of the most relevant vasculitis-promoting factors triggered by *T. cruzi* infection is endothelin-1 (ET-1). Much of the evidence suggests that this 21-amino acid peptide, produced by cardiac and endothelial cells among others, plays a pivotal role in the development of Chagas heart disease.^5^ ^-^ ^7^ During the progression of parasite-driven cardiomyopathy, increased levels and activity of ET-1 may result in vascular injury, platelet aggregation, cardiac remodelling, and enhanced secretion of inflammatory mediators that can adversely affect the myocardium.^5^ ^,^ ^8^ ^,^ ^9^

Given the well-demonstrated relationship between inflammation and Chagas pathogenesis, anti-inflammatory therapy is receiving increasing attention to improve cardiovascular function in established heart disease caused by *T. cruzi*.^10^ In this context, recent reports from different research groups, including our own, have shown that the anti-inflammatory and cardioprotective properties of curcumin (Cur), a natural polyphenolic flavonoid isolated from the rhizomes of *Curcuma longa*, are beneficial to attenuate cardiac injury caused by this pathogen.^11^ ^-^ ^13^ Current data further support the administration of Cur to improve vascular health.^14^ Nevertheless, additional research is needed to determine the precise mechanisms involved in its therapeutic action against endothelial dysfunction. In this study, our goal was to evaluate the effects of oral therapy with Cur on *T. cruzi*-mediated cardiovasculopathy in acutely infected mice and analyse the *in vitro* response of parasite-infected human microvascular endothelial cells treated with this natural compound.

MATERIALS AND METHODS

*Cell culture and infection* - Human microvascular endothelial cells (HMEC-1, Center for Disease Control and Prevention, Atlanta, GA, USA) were maintained in MCDB-131 medium (Sigma-Aldrich, St. Louis, MO, USA) supplemented with 2 mM L-glutamine, 1 μg/mL hydrocortisone hemisuccinate, 10 ng/mL recombinant human epidermal growth factor, 50 U/mL penicillin, 50 μg/mL streptomycin, and 10% foetal bovine serum (Sigma-Aldrich, St. Louis, MO, USA). Cells were cultured at 37ºC in 5% CO_2_. HMEC-1 were not used beyond passage 7.

The vascular endothelial cells were infected for different intervals (4-24 h) with *T. cruzi* trypomastigotes (cell:parasite ratio 1:5), Tulahuen strain, in the presence and absence of Cur at a final concentration of 13.5 or 27.0 μM (Fig. 1A). Cur (lot number 079K1756, purity ≥ 94% curcuminoids and ≥ 80% curcumin by high performance liquid chromatography) was purchased from Sigma-Aldrich. Endotoxin levels in the Cur preparations were undetectable (< 6.0 pg), as determined using a *Limulus* amoebocyte lysate analysis kit (Whittaker MA Bioproducts, Walkersville, MD, USA). In some experiments, HMEC-1 were incubated for 1 h in the presence and absence of ciclosporin A (CsA, 5 μM, Selleckchem, Munich, Germany) or Src kinase inhibitor-1 (Src-I1, 20 μM, Enzo Life Sciences, Lausen, Switzerland) before infection.

*Quantitative reverse transcription polymerase chain reaction (qRT-PCR)* - Total RNA was extracted from HMEC-1/myocardial cells and treated with DNase I using the RNeasy^®^ Micro kit (Qiagen, Hilden, Germany). mRNA levels of human and murine *ET-1* were determined by qRT-PCR using cDNA, obtained from the reverse transcription reactions, as template, with MyiQ^™^ Single-Color Real-Time PCR Detection System (Bio-Rad, Hercules, CA) and HotStart-IT^®^ SYBR^®^ Green One-Step qRT-PCR Master Mix Kit (Affymetrix, Santa Clara, CA, USA). The primer sequences that were used are as follows: human ET-1 forward, 5′-GCTCGTCCCTGATGGATAAA-3′ and reverse 5′-ATTCTCACGGTCTGTTGCCT-3′; mouse ET-1 forward, 5′-TCCTCTGCCCGTCTGAACAAGAAA-3′ and reverse 5′-GCCATCAGCAATAGCATCAAGGCA-3′; 18S forward, 5′-TCAAGAACGAAAGTCGGAGG-3′ and reverse, 5′-GGACATCTAAGGGCATCAC-3′.

Measurements of mouse pro-inflammatory cytokine (IL-6 and TNF-α) transcripts were also accomplished by qRT-PCR as described previously.^15^ 18S rRNA served as an internal control to normalise the amount of cDNA present in each sample. The data were analysed using the comparative difference in cycle number (ΔCT) method according to the manufacturer’s instructions.

*Measurement of endothelin-1 by enzyme-linked immunosorbent assay (ELISA)* - ET-1 levels were determined in mouse serum samples collected on day 14 of infection/treatment, and 24 h supernatants from HMEC-1 cultures, using the corresponding Quantikine ELISA Kit (R&D Systems, Abingdon, UK), according to the manufacturer’s guidelines. The threshold of sensitivity of the assay was 0.4 pg/mL.

*Calcium determination by fluorescent dye* - Parasite-induced changes in intracellular calcium concentration (ENT#091;Ca^2+^ENT#093;_i_) in human endothelial cells were detected using the Ca^2+^-sensitive indicator Fura-2/AM as described.^16^ HMEC-1 loaded with 1 μM Fura-2/AM were incubated in the presence and absence of Cur at increasing concentrations and infected with *T. cruzi* trypomastigotes immediately thereafter. Cells treated with Cur only were used as controls. At the indicated times, the fluorescence signal was recorded with a spectrofluorometer, with excitation and emission wavelengths at 340 and 510 nm, respectively.

*Immunoblot analysis* - For western blotting experiments, subcellular fractions from *T. cruzi*-infected vascular endothelial cells were generated as reported previously.^17^ The purity of the fractions was verified by analysing marker proteins, including α-tubulin (cytoplasmic), and proliferating cell nuclear antigen (PCNA, nuclear). Immunoblotting was carried out as described elsewhere.^16^ Uninfected and infected HMEC-1, with or without Cur treatment, were disrupted; solubilised extracts (20 μg) were separated on 6% sodium dodecyl sulphate-polyacrylamide gels, and then transferred to nitrocellulose membranes. After blocking, the membranes were probed for 2 h at 37ºC with rabbit polyclonal antibody against the c1 isoform of nuclear factor of activated T cells (NFATc1, 1:200, Santa Cruz Biotechnology, Dallas, TX, USA). The membranes were washed and incubated with secondary antibody (goat anti-rabbit IgG coupled to horseradish peroxidase; Jackson ImmunoResearch, West Grove, PA, USA) at 1:10,000 dilution, and the protein bands were visualised by a chemiluminescent peroxidase substrate (Amersham Pharmacia, Piscataway, NJ, USA).

**Fig. 1:**
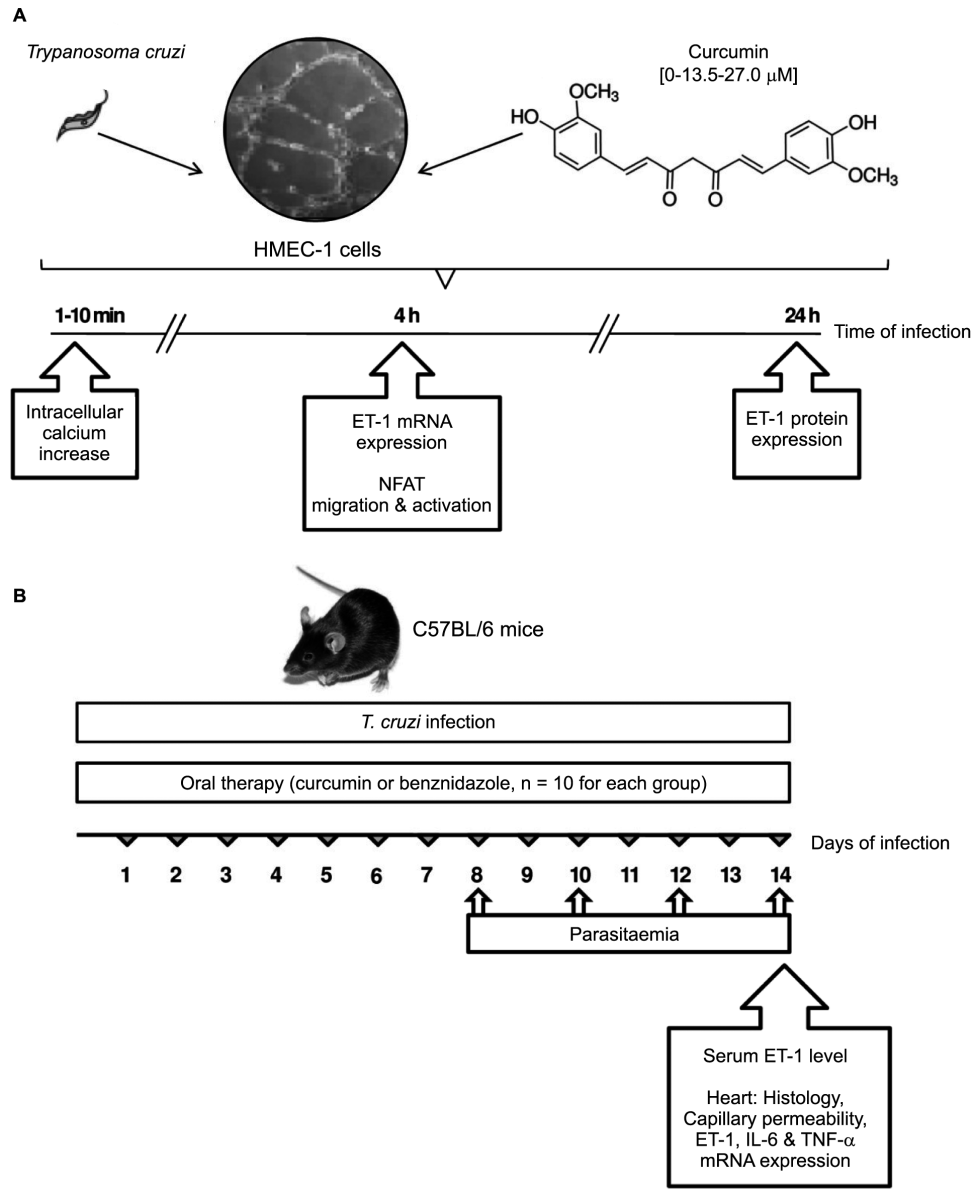
experimental design. (A) *In vitro* evaluation of the therapeutic effect of curcumin (Cur, 0, 13.5, 27.0 µM) on *Trypanosoma cruzi* infection of human microvascular endothelial cells (HMEC-1). Intracellular calcium increase, NFAT activation, and endothelin-1 (ET-1) production were analysed at different times of infection. (B) *In vivo* evaluation of oral Cur therapy in C57BL/6 mice acutely infected with *T. cruzi*. Comparative analysis was made with infected mice receiving benznidazole, a reference standard drug against the parasite, or no drug treatment. Parasitaemia was measured at 8, 10, 12, and 14 days of infection. Mice were euthanised on day 14 post-infection, and hearts and serum specimens were obtained for histology, studies of vascular integrity, and expression of pro-inflammatory mediators.

*Transfection and luciferase assays* - NFAT transcriptional activity was measured using an NFAT luciferase reporter ENT#091;p(NFAT)_3_-lucENT#093; containing three tandem copies of the NFAT response element fused to the IL-2 minimal promoter (a gift from M Fresno, Centro de Biología Molecular, Madrid, Spain). HMEC-1 were transfected essentially as described previously.^18^ Briefly, exponentially growing cells (4 × 10^4^/well) were incubated with a mixture of 0.5 mg of the reporter plasmid and Lipofectamine^®^ LTX-containing Opti-MEM supplemented with PLUS™ Reagent (Thermo Fisher Scientific, Waltham, MA, USA). In some experiments, the cells were co-transfected with 0.5 mg of the pSH102CD418 expression vector encoding an NFATc1 deletion mutant (1-418) that functions as a dominant negative for all NFAT isoforms (dnNFAT, kindly provided by MA Iñiguez, Centro de Biología Molecular, Madrid, Spain).^16^ The total amount of DNA in each transfection was kept constant by adding empty expression vector. Complete medium was then added to the cells and incubated at 37ºC for 18 h. The transfected HMEC-1 were exposed to *T. cruzi* parasites (or not) without or with Cur at increasing concentrations, as indicated. The inhibitory effect of 1-h CsA pretreatment of the cells on NFAT transcriptional activity was also studied. The cells were harvested and lysed, and luciferase activity was measured by using a luciferase assay system with a luminometer (Monolight 2010, Analytical Luminescence Laboratory, Ann Arbor, MI, USA). The transfection experiments were performed in triplicate. The luciferase activity data are presented as fold-induction ENT#091;experimental relative luciferase units (RLU)/basal RLU in absence of any stimulusENT#093;. The results were normalised to extract protein concentrations measured with a Bradford assay kit.

*Infection of mice* - The Tulahuen strain of *T. cruzi* was maintained by weekly intraperitoneal inoculation of Swiss mice. Eight-week old C57BL/6 male mice (n = 30; Jackson Laboratories, Bar Harbor, ME, USA) were infected intraperitoneally with 103 blood trypomastigotes, keeping a group of ten uninfected animals as controls. Infected mice were divided into three sub-groups: 10 of them were treated with Cur, another 10 were treated with benznidazole (Bz), and the remaining 10 mice received phosphate-buffered saline (PBS). Acute infection was verified by the detection of parasites via direct blood examination at 8, 10, 12, and 14 days post-infection (dpi). Mice were euthanised on day 14 post-infection, and hearts and serum specimens were collected for histology and *ET-1* mRNA and protein analyses (Fig. 1B).

*Cur treatment* - One subset of ten chagasic mice was treated with Cur dissolved in corn oil (100 mg/kg body weight), administered daily by oral gavage from day 1 through day 14 of infection (Fig. 1B). The dose was chosen based on previous oral studies of the anti-inflammatory effect of Cur in murine *T. cruzi* infection.^11^ ^,^ ^13^ Another set of ten infected mice received oral therapy with Bz at 100 mg/kg body weight/day over the same period.^19^ Bz (lot number 1614, purity ≥ 99% by high performance liquid chromatography), a reference standard drug against *T. cruzi*, was supplied by Laboratorio Elea, Buenos Aires, Argentina. Ten infected mice received PBS.

**Fig. 2:**
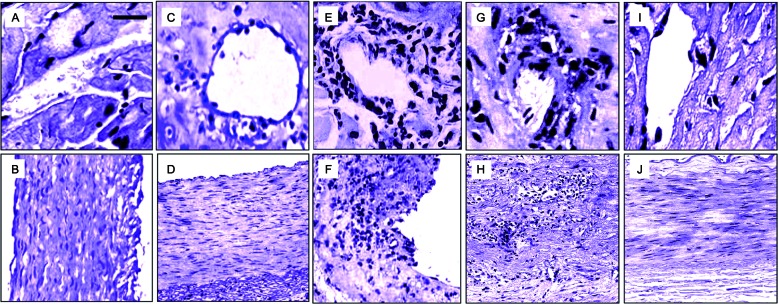
curcumin (Cur)-based therapy ameliorates cardiovascular pathology in mice with acute *Trypanosoma cruzi* infection. Heart specimens from *T. cruzi*-infected and uninfected C57BL/6 mice, orally treated with or without Cur or benznidazole (Bz) at 100 mg/kg body weight/day, were obtained. Three independent infections were conducted under the same conditions, each performed with ten mice per group. Microphotographs of histological analysis (haematoxylin and eosin staining) of cardiac tissues collected at 14 dpi are displayed. Top panels show heart vessels (A and C, 400X magnification) and aorta (B and D, 200X magnification) from uninfected mice, receiving (C-D) or not receiving (A-B) oral treatment with Cur. Scale bar is 100 μM. Bottom panels are representative images of heart distributing arteries (E, G and I, 400X magnification) and aorta (F, H and J, 200X magnification) from *T. cruzi*-infected mice, receiving (G-J) or not receiving (E-F) oral treatment with Bz (G-H) or Cur (I-J).

*Histological analysis of heart* - Mouse cardiac tissues were bisected and placed in 10% neutral-buffered formalin for at least 4 h at room temperature, followed by overnight incubation in 70% ethanol. Samples were then embedded in paraffin and semi-serial 5-μm tissue sections were prepared. Sections were deparaffinised, rehydrated, stained with haematoxylin and eosin, and examined by light microscopy at 200X and 400X magnification. A double-blind evaluation of the specimens was performed on randomised, precoded slides in a systematic fashion. For each animal, three myocardial sections were analysed, examining at least twenty areas per section (60 microscopic fields examined). For the heart vessels ENT#091;conduit (aorta) and distributing (medium- and small-sized) arteriesENT#093;, the inflammation score was graded as follows: 0 for no inflammation or minimal number of inflammatory cells; 1 for mild diffuse inflammatory infiltration or focal findings, involving < 25% of the vessel circumference; 2 for moderate inflammatory infiltration or focally marked, involving 25-50% of the vessel circumference; and 3 for heavy inflammatory infiltration or focally marked, involving > 50% of the vessel circumference.^20^

*Capillary permeability* - Capillary permeability was measured using an Evans-blue assay, as reported previously.^21^ The dye was extracted from the cardiac tissue by incubation with formamide (70ºC for 24 h) and the concentration of Evans blue (expressed as μg dye per gram tissue) was estimated by dual-wavelength spectrophotometry (620 and 740 nm).

*Statistical data evaluation* - Statistical analysis was performed using GraphPad Prism 5.0 software. Arithmetic means and standard deviations of the means were calculated. Significant differences among groups were assessed by using one-way analysis of variance followed by Tukey’s *post-hoc* tests. A value of p < 0.05 was considered significant.

*Ethics statement* - This study was carried out in strict accordance with the recommendations of the Committee on the Ethics of Animal Experiments from Universidad Nacional de San Luis (UNSL, San Luis, Argentina), following the recommendations of the Guide for the Care and Use of Laboratory Animals of the National Institutes of Health (NIH Publications No. 8023, revised 1978; Rockville, MD, USA). The protocol was approved by the Institutional Committee on the Ethics of Animal Experiments from UNSL (report #017-2405). All mice were maintained under pathogen-free conditions in our animal facilities. The individual room temperatures were kept at 18-22ºC with food and water *ad libitum*. A laboratory animal veterinarian was responsible for the care of the mice. The mice were euthanised in a CO_2_ chamber, and all efforts were made to minimise suffering.

**Fig. 3:**
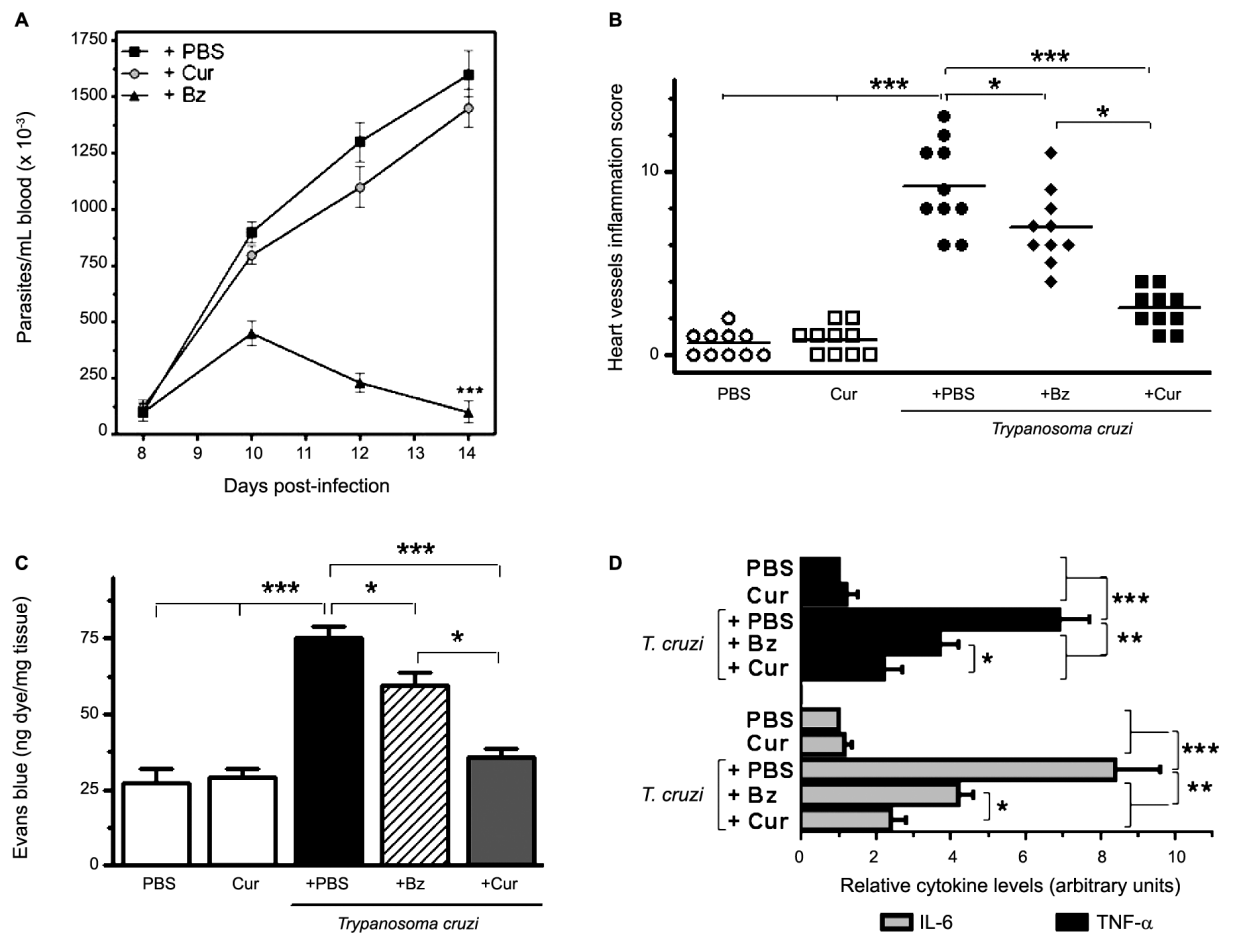
effects of curcumin (Cur) treatment on parasitaemia, vascular inflammation and permeability, and myocardial pro-inflammatory cytokine levels in acutely infected mice. (A) Parasitaemia was quantified by counting the number of circulating trypomastigotes in 5 μL of fresh blood collected from the tail vein at 8, 10, 12, and 14 days of infection. Results from infected mice receiving Cur, benznidazole (Bz), or phosphate-buffered saline (PBS) are depicted. ^***^p < 0.001 *versus* the remaining groups. (B) Inflammatory cell infiltration of heart vessels was histologically graded in tissue sections from the different uninfected and infected groups and expressed as a score, as described in Materials and Methods. ^*^p < 0.05 and ^***^p < 0.001 between indicated groups. (C) Vascular permeability was measured by Evans blue dye assay. ^*^p < 0.05 and ^***^p < 0.001 between indicated groups. (D) *IL-6* and *TNF-α* mRNA levels were analysed by quantitative reverse transcription polymerase chain reaction (qRT-PCR) in total heart extracts from uninfected and infected (14 dpi) mice, treated with PBS, Cur, or Bz. ^*^p < 0.05, ^**^p < 0.01, and ^***^p < 0.001 between indicated groups. Values are expressed as means ± standard deviations from three independent infections conducted under the same conditions, each performed with ten mice per group.

RESULTS

*Cur-based therapy ameliorates cardiovascular pathology in acutely T. cruzi-infected mice* - Mice bred on a C57BL/6 background, when infected with the Tulahuen strain of *T. cruzi*, are known to harbour abundant intracellular parasites, exhibiting disseminated myonecrosis and severe microvascular compromise in cardiac tissue.^5^ In our hands, intense vasculitis of the aorta, as well as perivasculitis of the medium- and small-sized myocardial arteries, was observed by day 14 of infection. Accumulation of inflammatory cells in the muscle tissue, in the aortic tunica media, and surrounding the vessel walls was evident in heart sections from acutely infected mice, whereas uninfected controls showed no histological signs of cardiomyopathy, aortitis, or perivascular trauma (Fig. 2A-B, E-F). We next addressed whether the anti-inflammatory properties of Cur can modulate *T. cruzi*-elicited vasculopathy. Treatment of acute Chagas mice with Bz at a standard dose (100 mg/kg/d) attenuated inflammation of the heart vasculature, although some inflammatory infiltrates were observed (Fig. 2G-H). Cur administered to uninfected mice for 14 days did not affect the histological features of their vessels (Fig. 2C-D). Along with the lack of adverse effects, oral Cur therapy (100 mg/kg/d) of recently infected mice substantially reduced inflammation of the myocardial arteries (Fig. 2I-J) without modifying the bloodstream parasite burden (Fig. 3A). In contrast, treatment with Bz led to a significant (p < 0.001) drop in parasitaemia (Fig. 3A). The survival rate of untreated, Cur-treated, and Bz-receiving mice was 100% after 14 days of *T. cruzi* infection. The total vessel inflammation score was significantly (p < 0.001) lower in hearts harvested from Cur-treated infected mice compared to that from mice injected with trypomastigotes only (Fig. 3B). To confirm these observations, we further verified the occurrence of disrupted vascular integrity in our experimental model. Using an Evans blue assay, we examined whether oral administration of Cur could interfere with altered capillary permeability in the myocardium after 14 days of infection. Evans blue dye, when injected into the bloodstream, binds to serum proteins, with the majority binding to albumin. If changes in vascular permeability occur, then the dye leaks from the vascular lumen into the interstitial tissue. The dye in the circulation is then washed out, and the tissues can be visually inspected for dye leakage. Treatment of parasite-harbouring mice with Cur led to a significant (p < 0.001) improvement in vascular integrity in the heart when compared with that of untreated, infected mice (Fig. 3C). Moreover, Cur-based therapy caused a remarkable (p < 0.01) downregulation in cardiac pro-inflammatory cytokine (*IL-6*, *TNF-α*) mRNA expression (Fig. 3D). At the doses used in this study, the early anti-inflammatory effect of Cur on the heart vasculature was as good as or even better than that achieved with Bz (Fig. 3B-D). Taken together, these results show that oral administration of Cur attenuates *T. cruzi*-dependent cardiovasculopathy during acute murine infection.

**Fig. 4:**
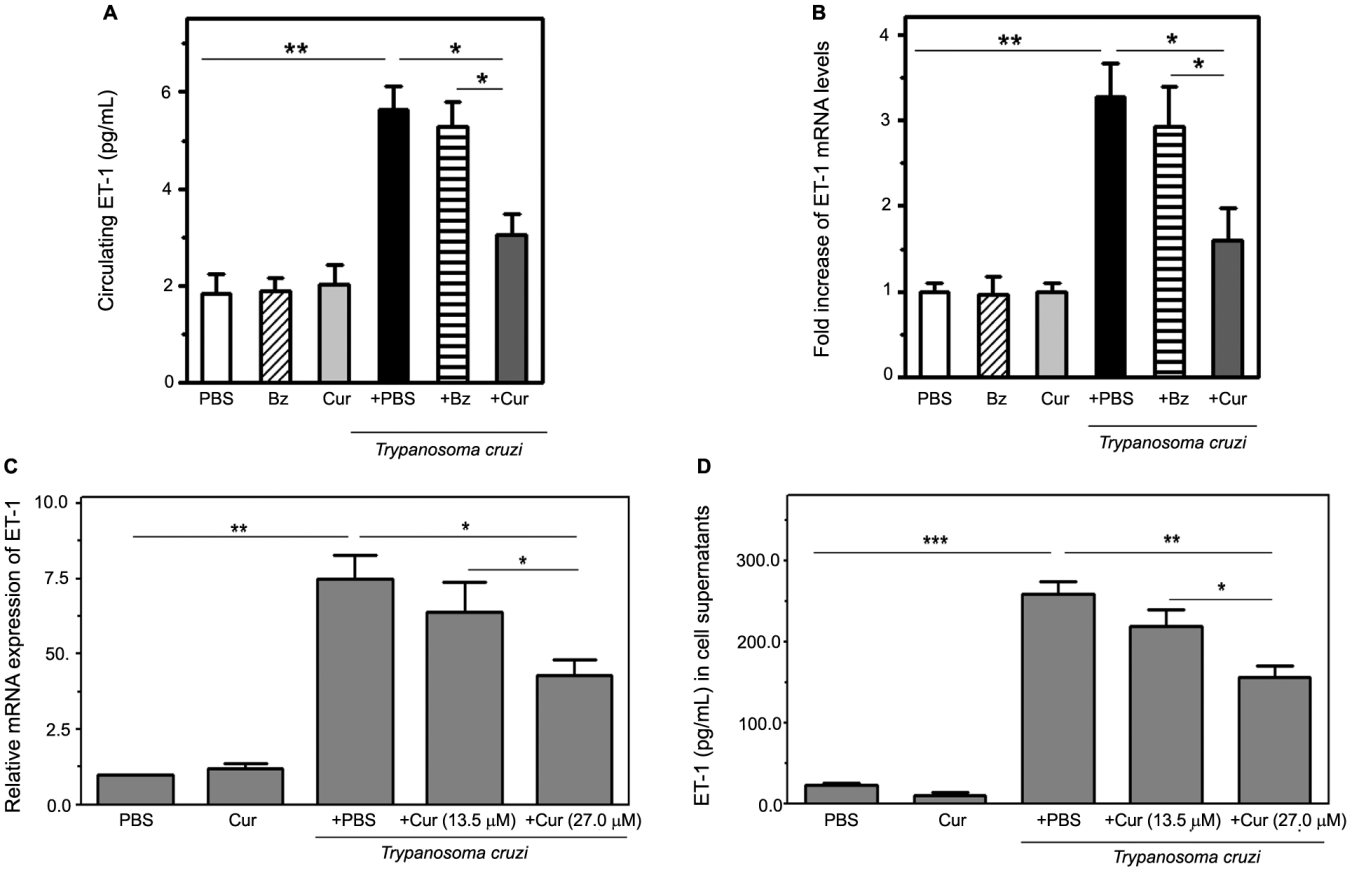
curcumin (Cur) attenuates endothelin-1 (ET-1) induction triggered by *Trypanosoma cruzi* infection. Top panels: ET-1 production was measured in *T. cruzi*-infected and uninfected mice, receiving or not receiving oral Cur/benznidazole (Bz) therapy (ten mice per group). (A) Circulating ET-1 concentration was determined by enzyme-linked immunosorbent assay (ELISA) in serum samples collected from experimental groups treated with Cur, Bz, or phosphate-buffered saline (PBS). (B) RNA isolated from heart tissue at 14 dpi was used to perform quantitative reverse transcription polymerase chain reaction (qRT-PCR) with specific primers, and normalised to ribosomal 18S RNA as described in Materials and Methods. Differences in *ET-1* mRNA levels among the groups are expressed as fold increase. Values are presented as means ± standard deviations from three independent infections performed with ten mice per group. ^*^p < 0.05 and ^**^p < 0.01 between indicated groups. Bottom panels: Dose effect of Cur on *ET-1* mRNA (C) and protein (D) expression triggered by *T. cruzi* infection of cultured human microvascular endothelial cells (HMEC-1). Cultures were parasite-infected (*T. cruzi*) *in vitro* for 4 h (for mRNA analysis) or 24 h (for soluble peptide measurement) in the presence (+ Cur, 13.5 or 27.0 μM) or in the absence (+ PBS) of phytochemical. Non-infected cell preparations, either treated with PBS or the flavonoid at 27.0 μM (Cur), were included in the assays. Data are the means ± standard deviations of three independent experiments, each performed in triplicate. *p < 0.05; **p < 0.01; ***p < 0.001, between indicated groups.

*Cur attenuates ET-1 induction triggered by T. cruzi infection* - Upon parasite infection (14 dpi), an elevated serum concentration of ET-1, an important contributor to vascular compromise that is characteristic of *T. cruzi*-mediated cardiomyopathy^6^ ^,^ ^22^, was demonstrated by ELISA in acute Chagas mice (Fig. 4A). This increase was paralleled by a three-fold enhanced *ET-1* mRNA expression in the hearts of this group (Fig. 4B). In contrast, Cur-treated infected animals displayed a significant (p < 0.05) decline in myocardial transcripts and circulating levels of the ET-1 peptide compared with those measured in the untreated infected controls and in the acutely infected mice under Bz therapy (Fig. 4A-B).

In addition, we tested the ability of Cur to inhibit *T. cruzi*-dependent generation of ET-1 by infected vascular endothelial cells.^23^ The turmeric-derived chemical produced a concentration-dependent inhibition of *ET-1* mRNA and protein expression in HMEC-1 infected with *T. cruzi* trypomastigotes *in vitro*. Increased levels of *ET-1* mRNA (4-h infection) and soluble peptide (24-h infection) were respectively detected by qRT-PCR and ELISA in cell extracts and media from infected microvascular cells (Fig. 4C-D). Treatment with Cur at 27.0 μM, but not 13.5 μM, significantly (p < 0.05 for transcript and p < 0.01 for peptide analysis) attenuated the ET-1 upregulation upon parasite invasion. These observations indicate that the concentration-dependent effect of Cur on the vascular endothelium may interfere with the early production of the vasoconstrictor, ET-1 peptide, which is stimulated over the course of acute Chagas disease.

*Cur impairs ET-1 production in T. cruzi-infected HMEC-1 through downregulation of the Ca* ^*2+*^ *-dependent NFAT signalling pathway* - ET-1 biosynthesis in epithelial and endothelial cells is regulated through specific intracellular signal transduction pathways, including those mediated by Ca^2+^-sensitive NFAT and c-Src kinase.^24^ ^,^ ^25^ Therefore, we investigated the involvement of these pathways in *T. cruzi*-induced ET-1 release from infected HMEC-1 using chemical inhibitors of each pathway. Increased ET-1 levels in the media from parasite-infected HMEC-1 were significantly (p < 0.05) attenuated by pretreatment of the cells with CsA, which blocks calcineurin-dependent NFAT activation (Fig. 3A). Contrariwise, specifically inhibiting Src kinase with Src-I1^25^ did not alter *T. cruzi*-induced ET-1 secretion. Addition of Cur to the infected endothelial cells reduced the release of the vasoactive peptide in a concentration-dependent manner by up to 46% at 27.0 μM Cur (p < 0.05) (Fig. 5A). The HMEC-1 from all cultures retained viability after incubation with the different inhibitors ENT#091;Supplementary data (Figure)ENT#093;. These findings suggest that Cur downregulates ET-1 secretion from microvascular endothelial cells in response to chagasic infection by interfering with Ca^2+^/calcineurin/NFAT interaction.

It has been demonstrated that *T. cruzi* infection induces a sustained elevation in ENT#091;Ca^2+^ENT#093;_i_ in human endothelial cells. We found that addition of Cur at 27.0 μM reduced the ENT#091;Ca^2+^ENT#093;_i_ response in *T. cruzi*-harbouring HMEC-1 to the basal levels recorded in uninfected cells treated with Cur only. This inhibitory effect could not be achieved using 13.5 μM Cur (Fig. 5B). Coupled with NFAT elevation in HMEC-1, different stimuli may also lead to calcineurin-mediated NFAT dephosphorylation and translocation to the nucleus.^26^ Immunoblot analysis demonstrated that *T. cruzi* infection induces NFATc1 activation in vascular endothelial cells (Fig. 5C). However, cell treatment with Cur at increasing doses (13.5-27.0 μM) prevented parasite-triggered NFATc1 nuclear translocation, thereby resulting in a progressive accumulation of cytoplasmic NFATc1 protein (Fig. 5C).

To further examine the cascade of NFAT activation promoted by *T. cruzi* in HMEC-1, we co-transfected the p(NFAT)_3_-luc reporter, carrying three NFAT response elements, with or without a dominant-negative dnNFAT plasmid. The expression of dnNFAT abrogated the pathogen-induced transcription of the reporter, supporting the involvement of NFAT in downstream signalling of the elevated ENT#091;Ca^2+^ENT#093;_i_ in infected cells (Fig. 5D). A similar effect was observed in HMEC-1 treated with CsA, a potent inhibitor of calcineurin-dependent NFAT induction. More importantly, Cur treatment significantly (p < 0.05 at 27.0 μM) attenuated the increase in NFAT-dependent luciferase activity in response to infection. Collectively, the above results show that Cur is capable of abolishing the ENT#091;Ca^2+^ENT#093;_i_-associated activation of the NFATc1 isoform in vascular endothelial cells infected by *T. cruzi*.

DISCUSSION

During Chagas disease progression, the severity of chronic myocardial involvement appears to be related to the magnitude of acute heart injury.^1^ ^,^ ^2^ Recently, we showed that Cur, the primary therapeutic curcuminoid derived from turmeric, can be effective in attenuating Chagas myocarditis.^13^ In this study, we further found that *in vivo* oral treatment with Cur dampens cardiovasculopathy in mice recently infected with *T. cruzi*. The decrease in inflammation of heart vessels and vascular permeability observed with Cur-based therapy is most likely attributable to its downregulating effect on different inflammatory mediators, rather than direct trypanocidal activity *in vivo*. No significant variation in the number of bloodstream parasites between mice with and without Cur treatment was recorded. Previously reported data indicate that concentrations of Cur in mouse serum and tissues do not easily reach the parasiticidal level determined *in vitro* for this natural product.^11^ ^,^ ^12^ Nevertheless, Cur might contribute to an adjunct therapy as it has been demonstrated to improve the anti-*T. cruzi* activity of Bz-based treatment.^12^ In addition, Cur is known to interfere with the regulatory actions of *T. cruzi* infection on several cardiopathogenic pathways linked to exacerbated production of cytokines, adhesion molecules, eicosanoids, natriuretic peptides, leukotrienes, and reactive oxygen/nitrogen species.^11^ ^,^ ^13^

**Fig. 5:**
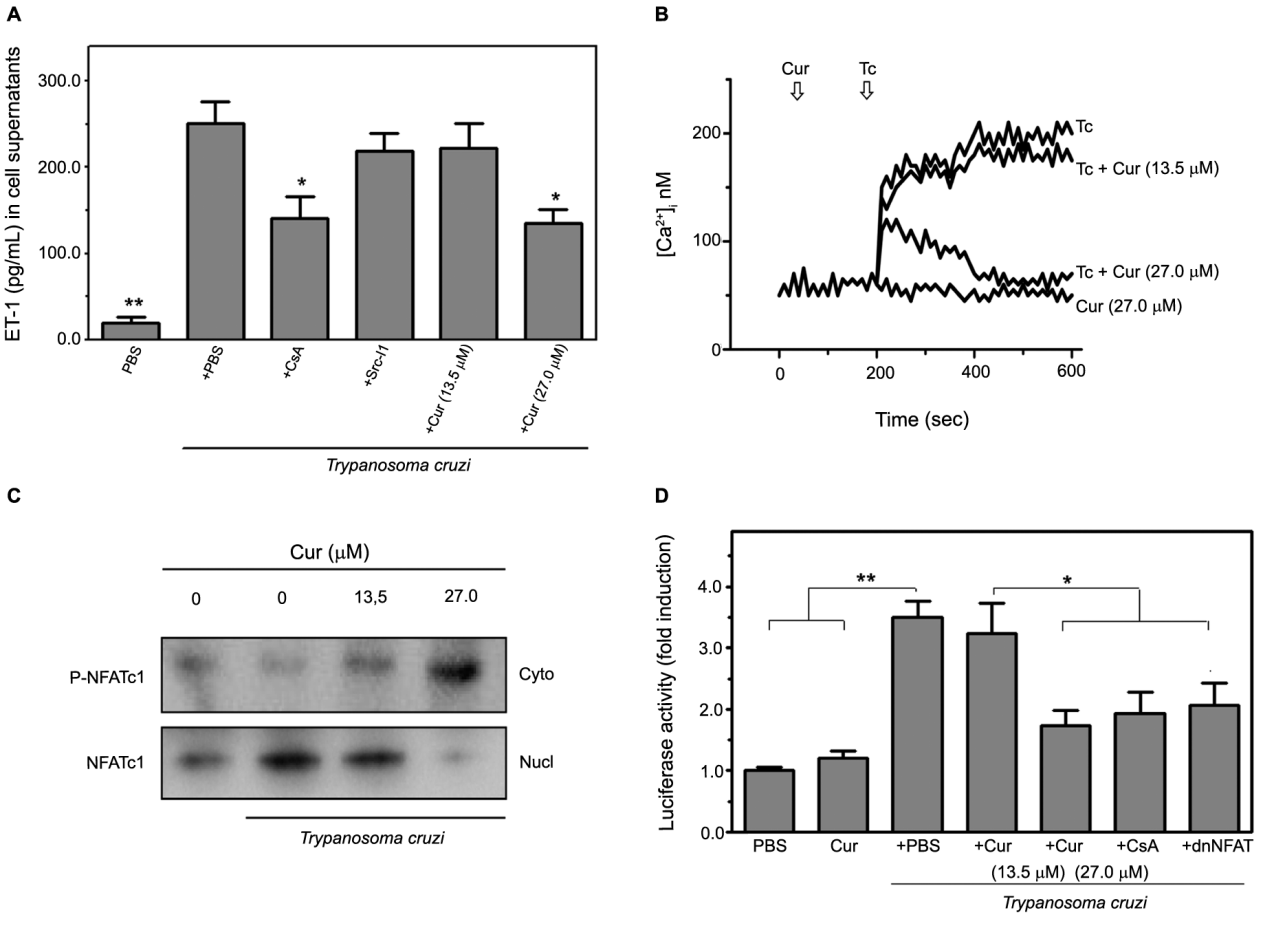
curcumin (Cur) impairs endothelin-1 (ET-1) production in *Trypanosoma cruzi*-infected human microvascular endothelial cells (HMEC-1) through downregulation of Ca^2+^-sensitive NFAT signalling. The involvement of Ca^2+^-dependent mechanisms in *T. cruzi*-promoted ET-1 release from infected HMEC-1 was verified. (A) Soluble ET-1 levels in the media of uninfected (PBS) or infected (*T. cruzi*) cells, cultured in the presence or in the absence of Cur at different concentrations (13.5 and 27.0 μM), were measured by enzyme-linked immunosorbent assay (ELISA). In some experiments, the cells were pre-treated for 1 h with chemical inhibitors of NFAT or c-Src kinase-mediated pathways (5 μM CsA or 20 μM Src-I1, respectively), and their effect on ET-1 secretion was further determined. Data are the means ± standard deviations of three independent experiments, each performed in triplicate. *p < 0.05; **p < 0.01, *versus* untreated cells only infected with *T. cruzi*. (B) Calcium increase modulated by Cur was studied. HMEC-1 cells, exposed or not exposed to the parasite (Tc), were loaded with the Ca^2+^ indicator, Fura-2/AM, and changes in _i_ upon *T. cruzi* infection, performed in the presence or in the absence of Cur (13.5 or 27.0 μM), were recorded. Uninfected cells incubated with Cur at the highest dose only were used as a control. Arrows indicate the time (sec) when the parasite and/or the phytochemical were added. The results presented are representative of three independent experiments. (C) NFAT subcellular localisation was visualised by western blotting. HMEC-1 were infected with trypomastigotes, with or without addition of Cur at 13.5 or 27.0 μM, for 4 h. Fractionated extracts from both untreated and Cur-treated infected cells, as well as uninfected and untreated controls, were analysed. The phosphorylated cytosolic (P-NFATc1) or dephosphorylated nuclear (NFATc1) forms of the transcription factor and loading controls for cytosolic and nuclear proteins (α-tubulin and PCNA, respectively) are indicated. Cyto, cytosolic extracts; Nucl, nuclear extracts. One out of three separate experiments performed is shown. (D) The downstream cascade of NFAT activation promoted by *T. cruzi* in HMEC-1 was also examined. The cells were transiently transfected with the p(NFAT)_3_-luc reporter, carrying three NFAT response elements, alone or co-transfected with a dominant-negative NFAT (dnNFAT) plasmid. Cultures were then infected with the parasite (*T. cruzi*) and PBS or Cur at increasing doses (13.5, 27.0 μM) was added. In some experiments, HMEC-1 were treated with 5 μM CsA prior to infection. Transfected cells without treatment or trypomastigotes (PBS), or incubated with phytochemical only (Cur), were also assayed. Luciferase activity is expressed as fold induction (mean ± standard deviation) relative to that measured with empty vector-transfected cells. One out of three separate experiments performed is displayed. *p < 0.05; **p < 0.01 between indicated groups.

Another relevant molecular target for Cur intervention is ET-1, an important contributor to the vascular compromise that is characteristic of *T. cruzi*-mediated cardiomyopathy.^6^ ^,^ ^22^ A growing body of literature has demonstrated key changes in the vascular reactivity to ET-1 in certain pathological conditions. Particularly, this vasoactive peptide has been shown to play a major role in the development of vascular disruption caused by Chagas and other infectious diseases.^27^ In our series, acutely infected mice presented increased ET-1 levels in the circulation and cardiac tissue. *T. cruzi*-infected hosts frequently display elevated plasma levels of the peptide as well as augmented expression in the vasculature.^5^ ^,^ ^8^ ^,^ ^9^ Moreover, ET-1 is increasingly recognised as a pro-inflammatory cytokine that promotes vasoconstriction, vascular permeability, platelet aggregation, and heightened production of adhesion molecules and inflammatory agents, all of which have been tightly associated with Chagas cardiovasculopathy.^27^ These alterations further contribute to the development of the typical dilated cardiomyopathy observed in chronic symptomatic cases.^7^

Oral Cur therapy significantly reduced *T. cruzi*-dependent induction of ET-1 and pro-inflammatory cytokines (IL-6, TNF-α) in the infected mice. According to our experience, high-dose Cur administration seems to be more efficacious than the standard trypanocidal drug Bz in preventing early overproduction of heart-derived inflammatory mediators in infected mice. This regulatory effect accompanied the amelioration of vascular inflammation achieved by Cur treatment. In addition, we found that Cur particularly inhibits *T. cruzi*-dependent generation of ET-1 by infected vascular endothelial cells.^23^ Pharmacological interventions aimed at attenuating ET-1 synthesis and activities deserve consideration in the search for an effective strategy against cardiovascular inflammatory processes. Cur therapy has proven effective in modulating ET-1 levels and actions in both animal models and clinical trials.^28^ Our findings suggest that this regulatory ability could contribute to reducing *T. cruzi*-elicited vascular trauma.

We further demonstrated *in vitro* that Cur downregulates ET-1 secretion from microvascular endothelial cells in response to chagasic infection by interfering with Ca^2+^/NFAT-dependent signalling. To date, several *cis*-acting elements have been characterised within the ET-1 promoter. Of note, two NFAT consensus sites (GGAAA) located in the distal upstream region are capable of binding NFATc1/c3 isoforms and participating in transcriptional regulation of ET-1 gene expression.^24^ The vascular endothelium is an important source of ET-1 upon *T. cruzi* infection.^5^ Since early studies^23^, a solid link has been established between parasite-stimulated synthesis and secretion of biologically active ET-1 from infected endothelial cells and subsequent vasospasms and cardiac remodelling. In the cardiovascular system, ET-1 is able to target different cell types and trigger multiple intracellular signalling pathways implicated in the pathophysiology of Chagas disease, including those mediated by elevated ENT#091;Ca^2+^ENT#093;_i_ levels coupled to nuclear translocation of dephosphorylated NFAT.^29^ Consistent with previously documented observations^30^, we recently described a protective action of Cur involving the suppression of pathogenic prostaglandin E_2_/natriuretic peptide production through inhibition of the NFAT pathway in *T. cruzi*-harbouring cardiomyocytes.^13^ In addition, our findings herein suggest that Cur significantly impairs ET-1 secretion from *T. cruzi*-infected vascular endothelium, at least in part, by blocking the Ca^2+^/calcineurin/NFATc1 cascade.

Trypomastigote-endothelial cell interactions are among the first to occur during the initial phase of *T. cruzi* infection and have been identified as a critical step in Chagas pathogenesis. Treatment of vascular and, in particular, microvascular dysfunction might be important to help mitigate not only Chagas heart disease but also other infectious and non-infectious cardiomyopathies. The results from this study unveil a putative mechanism of action of Cur involving inhibition of the Ca^2+^/NFAT-dependent secretion of the cardiopathogenic ET-1 peptide by *T. cruzi*-infected vascular endothelium. This targeted effect could partly account for the marked reduction in inflammatory injury observed in the myocardial vessels and tissues from acutely infected mice subjected to oral Cur therapy. Our current and previous^13^ findings provide new perspectives for exploring the therapeutic potential of this multifunctional phytochemical to reduce parasite-driven vasculitis and myocarditis. Despite the morbidity and mortality inflicted by Chagas disease, etiologic treatment still relies on only two drugs, nifurtimox and Bz. Furthermore, due to the well- known toxicity and limited effects in prolonged symptomatic infection with the currently used drugs, research and development of novel, non hazardous medications with enhanced efficacy is urgently needed to treat the large population infected with this pathogen. Over the last decade, numerous molecules, both natural and synthetic, have been postulated for Chagas disease chemotherapy, but their unsuitable and undesirable structural and pharmacokinetic properties have precluded further development. Therefore, the potent anti-inflammatory and cardio/vasoprotective actions, low cost, advantageous oral administration, and outstanding safety profile evidenced by Cur deserve attention to highlight this phytochemical as a complementary tool to the limited array of therapies so far available for the treatment of Chagas cardiomyopathy.
